# Association between single-nucleotide polymorphism rs145497186 related to NDUFV2 and lumbar disc degeneration: a pilot case–control study

**DOI:** 10.1186/s13018-022-03368-y

**Published:** 2022-10-29

**Authors:** Ziyu Wang, Lu Chen, Qinghui Li, Hengshuo Zhang, Yu Shan, Linzeng Qi, Hongliang Wang, Yunzhen Chen

**Affiliations:** 1 Department of Orthopedics, Qilu Hospital of Shandong University, #107 Wenhuaxi Road, Jinan, 250012 Shandong People’s Republic of China; 2 Cheeloo College of Medicine, Shandong University, Jinan, 250012 Shandong People’s Republic of China

**Keywords:** NDUFV2, Single-nucleotide polymorphism, Lumbar disc degeneration, Visual analogue scale, Case–control study

## Abstract

**Objective:**

The association between the single-nucleotide polymorphisms (SNPs) rs28742109, rs12955018, rs987850, rs8093805, rs12965084 and rs145497186 related to gene named NADH dehydrogenase [ubiquinone] flavoprotein 2 (NDUFV2) and lumbar disc degeneration (LDD) was preliminary investigated in a small sample size.

**Methods:**

A total of 46 patients with LDD and 45 controls were recruited at Qilu Hospital of Shandong University, and each participant provided 5 mL peripheral venous blood. NA was extracted from the blood of each participant for further genotyping. The frequency of different genotypes in the case group and control group was determined, and analysis of the risk of LDD associated with different SNP genotypes was performed. The visual analogue scale (VAS) scores of the patients’ degree of chronic low back pain were calculated, and the relationship between VAS scores and SNPs was analysed.

**Results:**

After excluding the influence of sex, age, height, and weight on LDD, a significant association between SNP rs145497186 related to NDUFV2 and LDD persisted (*P* = 0.006). Simultaneously, rs145497186 was found to be associated with chronic low back pain in LDD populations.

**Conclusion:**

NDUFV2 rs145497186 SNP could be associated with susceptibility to LDD and the degree of chronic low back pain.

**Supplementary Information:**

The online version contains supplementary material available at 10.1186/s13018-022-03368-y.

## Introduction

Chronic low back pain (CLBP) is a common cause of reduced quality of life worldwide [1]. According to statistics, approximately 60–80% of people experience CLBP symptoms during their lifetime [2]. In-line with reports thus far, up to 42% of CLBP cases are related to lumbar disc disease [3]. There are two types of lumbar disc disease that lead to CLBP: secondary pain caused by lumbar disc herniation or lumbar spinal stenosis and lumbar disc degeneration (LDD), which is the main cause of CLBP [4].

The pathogenesis of LDD is complicated and is influenced by genetic factors, physical loading and other environmental factors [5]. Indeed, recent studies have shown that genetic factors, such as those related to mitochondrial respiration, autophagy, and apoptosis, play a significant role in LDD [6]. Among these factors, autophagy and apoptosis have been studied in previous research [7, 8]. Moreover, it has been shown that oxidative stress helps to maintain the homeostasis of nucleus pulposus cells by balancing autophagy and apoptosis [9]. However, there is no evidence that genovariation in mitochondrial respiration-related genes is associated with LDD.

The NDUFV2 gene participates in mitochondrial respiration, encoding NADH dehydrogenase [ubiquinone] flavoprotein 2 (NDUFV2), which is the core subunit of the mitochondrial membrane respiratory chain NADH dehydrogenase (Complex I) [10]. Using ubiquinone as an electron acceptor, it catalyses electron transfer from NADH through the respiratory chain [11]. Nevertheless, as the association between NDUFV2 abnormalities and LDD is not fully understood, further investigations are needed to elucidate their relationship.

As a very important aspect of genetic polymorphism, SNPs have been recognized as playing a critical role in disease susceptibility [12]. Many studies have shown the association between SNPs and susceptibility to common orthopaedic diseases [13], such as muscle injuries [14], tendon and ligament injuries [15, 16]. Furthermore, it is interesting that some studies have shown that these SNPs related to musculoskeletal diseases can affect athletic performance [17, 18]. Therefore, our study aimed to assess the association between NDUFV2 rs28742109, rs12955018, rs987850, rs8093805, rs12965084 and rs145497186 SNPs and LDD [19].

## Materials and methods

### Study subjects

A total of 91 participants were recruited at Qilu Hospital of Shandong University from March 2019 to May 2019, including 46 patients with degenerative lumbar disc herniation and 45 controls. This study was approved by the ethics committee of Qilu Hospital of Shandong University and was conducted according to the guidelines of the Declaration of Helsinki. All participants were aware of this research and signed the informed consent form [20]. General data, including sex, age, height, weight and body mass index (BMI), were collected [21].

### Inclusion and exclusion criteria

*Inclusion Criteria* 1. adults over 18 years old, 2. the case group was patients admitted for surgery who had symptoms of low back pain, and MRI showed that they suffered from LDD, 3. normal mental consciousness and able to actively cooperate with the researchers, and 4. voluntarily participated and agreed to sign informed consent.

*Exclusion Criteria* 1. a history of scoliosis, spinal deformities, metabolic diseases, cardiovascular diseases, and malignant tumours, among others, 2. a history of spinal trauma, 3. undergone operations at the same intervertebral disc, and 4. unable to cooperate with the researchers.

This study used these criteria as a reference to select participants.

### Genotyping

Genomic deoxyribonucleic acid (DNA) was extracted from peripheral venous blood and stored at − 80 °C until genotyping was performed [22, 23]. Capital Biotechnology Precision Medicine Research Array Kit (CBT-PMRA) (Thermo Fisher Scientific, Waltham, MA, USA), a specialized chip based on the Axiom 2.0 platform, was used for genotyping. The microarray experiments were carried out according to the official standard operating procedures (Axiom™ 2.0 assay 96-array format manual workflow user guide). After amplification, hybridization, ligation, staining, washing and array scanning, data were obtained for further research.

### Chronic low back pain VAS score assessment

Forty-six patients diagnosed with LDD were evaluated with the VAS: zero points, no pain; one to three points, mild pain not affecting sleep; four to six points, moderate pain mildly affecting sleep; seven to ten points, severe pain and unable to sleep [24].

### Statistical and bioinformatic analyses

SNPs related to NDUFV2 were detected via CBT-PMRA chips. A chi-square test was used to assess allele deviation from Hardy–Weinberg equilibrium (HWE) [25]. Then, the chi-square test was used to determine statistically significant SNPs. Analysis of a genetic association was estimated under five different models (allelic, dominant, recessive, heterozygous and homozygous) using SPSS 21.0 statistical software (SPSS Inc., Chicago, USA) [26]. In screening for SNPs with statistically significant differences, *P* values < 0.01 in two-tailed test were considered statistically significant, for other statistical analyses, *P* values < 0.05 were considered statistically significant. Linkage disequilibrium (LD) analysis was performed with Haploview and LD blocks [27], where the number shown in each block is one hundred times the D’ value. The statistical analysis was performed using the statistical computing software R [28].

To determine the functional consequences of target SNPs, RegulomeDB and 3D SNP databases were used. RegulomeDB annotates SNPs with known and predicted regulatory elements in the intergenic regions of the human genome [29]. 3D SNP is a database for linking human noncoding SNPs to three-dimensional interacting genes [30]. It comprehensively evaluates the role of SNPs based on six different functional categories, including 3D interacting genes, enhancer state, promoter state, transcription factor-binding sites, sequence motifs altered and conservation; the higher the score is, the stronger the role of the SNP in this function is [31].

## Results

### Characteristics of the study subjects

Statistical tests were conducted by collecting the basic information of the participants. As sex, age, height, weight, and BMI were not significantly different between the case and control groups (*P* > *0.05*) (Additional file 1: Table S1), these factors had no significant relationship with LDD.

The *P* values of the HWE test for the investigated SNPs were all greater than 0.05 (Additional file 2: Table S2). Therefore, it was believed that these SNPs in the research population satisfied the HWE and that the samples reflected the population [32].

There were six SNPs detected from the SNP chip, including rs145497186, rs28742109, rs12955018, rs987850, rs8093805 and rs12965084. The results of the relationship of these six SNPs between the case and control groups showed significant differences for rs8093805, rs12965084 and rs145497186 (Table 1). The relationship between the genotypes of these SNPs and LDD was analysed by the chi-square test (Table 2), with rs145497186 having a significant relationship with LDD.

**Table 1:** Chi-square Test of rs28742109, rs12955018, rs987850, rs8093805, rs12965084 and rs145497186 related to NDUFV2

SNP	Control group (n)^a^			Case group (n)^a^			*P* value
	11	12	22	11	12	22	
rs28742109	28	13	4	35	11	0	0.085
rs12955018	41	4	0	36	8	2	0.162
rs987850	26	18	1	36	10	0	0.086
rs8093805	18	20	7	31	11	4	0.032
rs12965084	18	20	7	29	15	2	0.048
rs145497186	45	0	0	39	7	0	**0.006**

Statistically significant differences *P* values < 0.01 are in bold

^*^SNP, single-nucleotide polymorphism

^a^11 presents common homozygote, 22 presents rare homozygote, 12 presents heterozygote, 1 presents common allele, 2 presents rare allele

**Table 2:** The relationship between SNPs related to NDUFV2 and LDD

Genotype^a^	Control group (n)		Case group (n)		${x}^{2}$ value	*P* value
*rs145497186*						
Allelic Model (1 vs. 2)	90	0	85	7	7.122	**0.008**
Dominant Model (11 vs. 12 + 22)	45	0	39	7	7.418	**0.006**
Recessive Model (11 + 12 vs. 22)	45	0	46	0	–	1.000
Heterozygous Model (11 vs. 12)	45	0	39	7	7.418	**0.006**
Homozygous Model (11 vs. 22)	45	0	39	0	–	0.944
*rs28742109*						
Allelic Model (1 vs. 2)	69	21	81	11	4.064	0.004
Dominant Model (11 vs. 12 + 22)	28	17	35	11	2.053	0.152
Recessive Model (11 + 12 vs. 22)	41	4	46	0	4.277	0.039
Heterozygous Model (11 vs. 12)	28	13	35	11	0.659	0.417
Homozygous Model (11 vs. 22)	28	4	35	0	4.653	0.031
*rs12955018*						
Allelic Model (1 vs. 2)	86	4	80	12	4.195	0.041
Dominant Model (11 vs. 12 + 22)	41	4	36	10	2.885	0.089
Recessive Model (11 + 12 vs. 22)	45	0	44	2	2.000	0.157
Heterozygous Model (11 vs. 12)	41	4	36	8	1.647	0.199
Homozygous Model (11 vs. 22)	41	0	36	2	2.214	0.137
*rs987850*						
Allelic Model (1 vs. 2)	70	20	82	10	4.259	0.039
Dominant Model (11 vs. 12 + 22)	26	19	36	10	4.396	0.036
Recessive Model (11 + 12 vs. 22)	44	1	46	0	1.034	0.309
Heterozygous Model (11 vs. 12)	26	18	36	10	1.501	0.221
Homozygous Model (11 vs. 22)	26	1	36	0	0.355	0.244
*rs8093805*						
Allelic Model (1 vs. 2)	56	34	73	19	6.464	0.011
Dominant Model (11 vs. 12 + 22)	18	27	31	15	6.867	0.009
Recessive Model (11 + 12 vs. 22)	38	7	42	4	1.007	0.316
Heterozygous Model (11 vs. 12)	18	20	31	11	5.877	0.015
Homozygous Model (11 vs. 22)	18	7	31	4	2.675	0.102
*rs12965084*						
Allelic Model (1 vs. 2)	56	34	73	19	6.464	0.011
Dominant Model (11 vs. 12 + 22)	18	27	29	17	4.837	0.028
Recessive Model (11 + 12 vs. 22)	38	7	44	2	3.206	0.073
Heterozygous Model (11 vs. 12)	18	20	29	15	2.865	0.091
Homozygous Model (11 vs. 22)	18	7	29	2	4.764	0.029

Statistically significant differences *P* values < 0.05 are in bold

^*^^a^11 presents common homozygote, 22 presents rare homozygote, 12 presents heterozygote, 1 presents common allele, 2 presents rare allele

### Distribution of the SNP rs145497186 genotype and its relationship with LDD

The genotypes of rs145497186 for the LDD patients and controls are listed in Table 2. The distribution of rs145497186 between the LDD patients and controls showed a statistically significant difference (*P* = *0.006*), and no deviation from HWE was observed in the control group (*P* = *0.930*) (Additional file 2: Table S2).

The genotype frequencies in the control group were 11 (100%), 12 (0%), and 22 (0%), and those in the case group were 11 (84.8%), 12 (15.2%), and 22 (0%). The results of the chi-square test revealed distribution differences in rs145497186 between the case and control groups in the allelic model (*P* = 0.008), dominant model (*P* = 0.006) and heterozygous model (*P* = 0.006) (Table 2).

### Relationship between single-nucleotide polymorphisms related to NDUFV2 and chronic low back pain

The 46 participants in the case group were divided into two groups based on VAS score: VAS < 4 and VAS $\ge$ 4 [33]. The groups did not differ significantly in terms of sex, age, height, weight, body mass index (BMI), spinal canal occupation ratio (SCOR), or decrease spinal canal ratio (DSCR) (Additional file 3: Table S3). Among all SNPs, rs145497186 was significantly different in the VAS score groups (group 1 included patients whose VAS scores were < 4; group 2 included patients whose VAS scores were $\ge$ 4) (Table 3).

**Table 3:** The relationship between SNP that related to NDUFV2 and CLBP VAS Score in case group

SNP	VAS < 4 (n)^a^			VAS $\ge$ 4 (n)^a^			*P* value
	11	12	22	11	12	22	
rs28742109	17	2	0	18	9	0	0.074
rs12955018	17	2	0	19	6	2	0.246
rs987850	16	3	0	24	3	0	0.929
rs8093805	15	3	1	16	8	3	0.373
rs12965084	15	4	0	14	11	2	0.133
rs145497186	13	6	0	26	1	0	**0.010**

*VAS* Visual Analogue Scale

Statistically significant differences *P* values < 0.05 are in bold

^*^SNP, single-nucleotide polymorphism

^a^11 presents common homozygote, 22 presents rare homozygote, 12 presents heterozygote, 1 presents common allele, 2 presents rare allele

### Linkage disequilibrium of the screened SNPs related to NDUFV2 in LDD

**Fig. 1:**
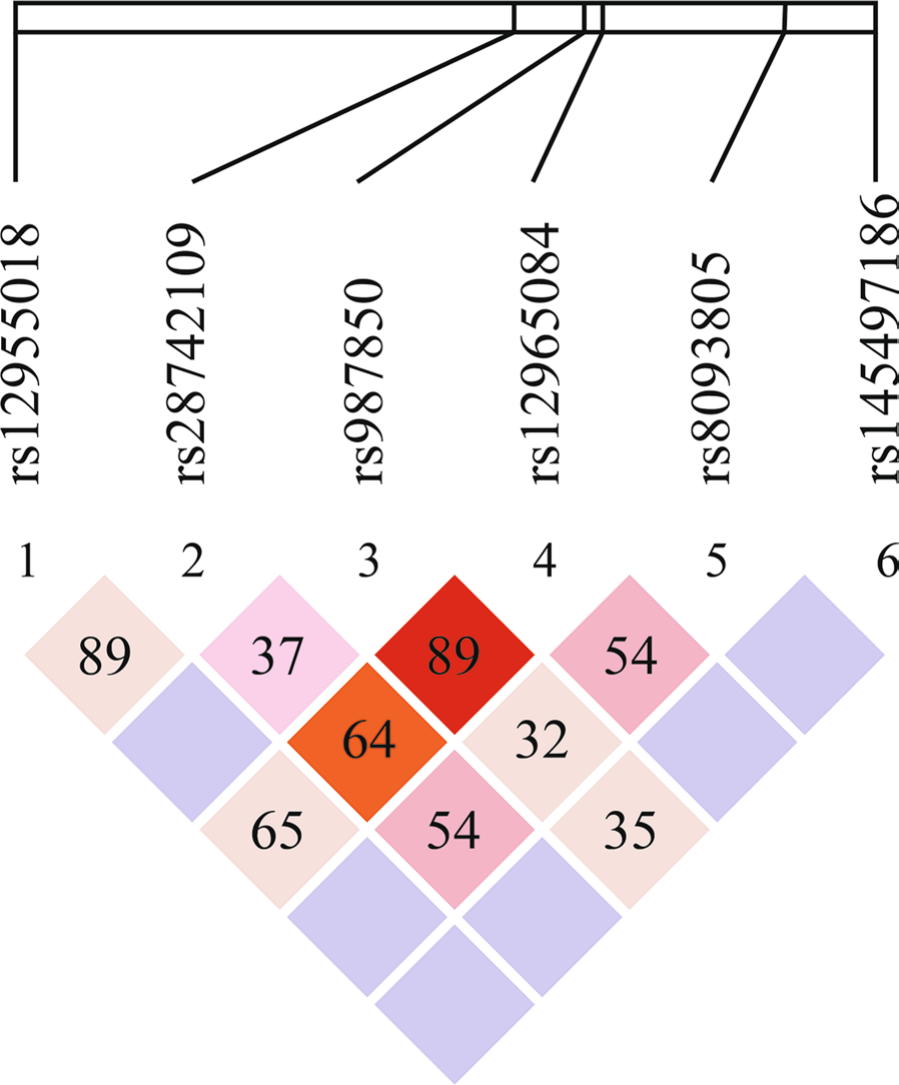
Linkage disequilibrium plot based on 6 SNPs related to NDUFV2. Values of ${r}^{2}$ for SNP pairs are indicated in each cell

LD among the six SNPs was low in general (Fig. 1). For rs987850 and rs12965084, the D’ value was 0.89, the ${r}^{2}$ value was 0.381, and a significant association signal was identified. However, the D’ value of rs145497186 and rs987850 was 0.355, and their ${r}^{2}$ value was 0.001, which were both the lowest in the LD plot.

### Functional consequences of SNP rs145497186

**Fig. 2:**
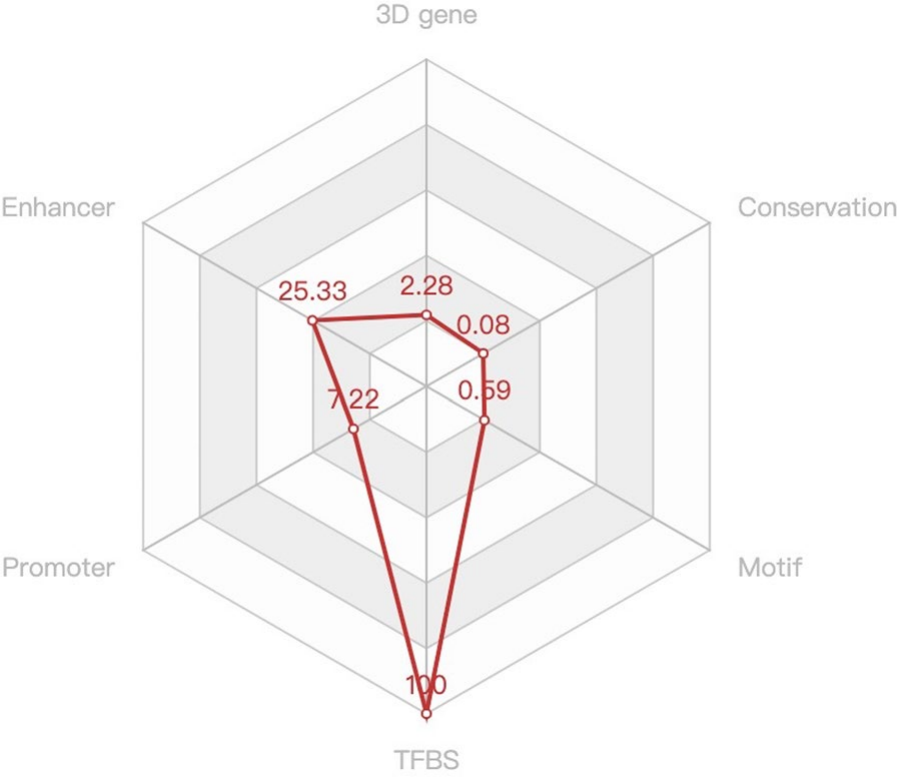
The scores of various functions of rs145497186 in 3D SNP

The SNP rs145497186 is located on chromosome 18, p13, with a rank score of 2b. RegulomeDB uses a rank scoring strategy to assess the functional significance of SNPs, whereby each SNP is assigned a rank score ranging from one to six, with a lower score indicating more functional significance [34]. Therefore, rs145497186 has obvious functional significance. As a noncoding SNP, the function of rs145497186 is completely shown in the 3D SNP in Fig. 2. The enhancer score was 25.33, which was the second highest of the six categories of scores. The score of transcription factor-binding site (TFBS) was 100, which was the highest score. The results showed SNP rs145497186 to be located in a region containing 205 transcription factor-binding sites.

## Discussion

Gene expression may be affected by various factors, including mutations caused by SNPs, which may alter the processes of transcription and translation [35]. Accordingly, changes in the function of the proteins produced may occur. These changes may directly or indirectly affect normal physiological activities and contribute to disease [36]. Therefore, it is very meaningful to explore the relationship between disease and SNPs.

The protein produced by the NDUFV2 gene is one of the core components of Complex I, and Complex I (Cx I) (NADH-ubiquinone oxidoreductase; EC 1.6.5.3) is an electron entry point in the mitochondrial respiratory electron transport chain (ETC) [37]. Relevant studies have shown that a low abundance of the protein NDUFV2 can reduce electron transport in the mitochondrial electron transport chain, thereby decreasing the production of reactive oxygen species and free radicals, which can prolong the lifespan of a cell [38]. The NDUFV2 gene has been mainly studied in mental diseases, brain diseases, prostate cancer and hypertrophic cardiomyopathy [39–42].

To the best of our knowledge, there have been no relevant studies about variation in NDUFV2 and its impact on disease in orthopaedics, especially in the field of lumbar disc degeneration. Therefore, the main goal of this study is to detect the relationship between SNPs related to NDUFV2 and LDD and to explore the relationship between SNPs of NDUFV2 and the genetic susceptibility of LDD disease, then provide a new potential target for the precision medicine of LDD. A total of 112 SNPs associated with NDUFV2were detected using a chip, six of which were significantly different and selected for follow-up analysis. Through data analysis, six related SNPs were identified: rs28742109, rs12955018, rs987850, rs8093805, rs12965084 and rs145497186. The genotype of these six SNPs in each study subject was detected. Then, five genetic models were assessed for each SNP, including the allele model, dominant model, recessive model, heterozygous model and homozygous model. The Hardy–Weinberg equilibrium test was used to identify that the subjects were representative of the population (Additional file 2: Table S2). Furthermore, statistical analysis showed no difference in sex, age, height, weight or BMI. After excluding the influence of these factors, a chi-square test was applied to examine the relationship between each SNP and LDD (Table 1).

The results showed the genotype of SNP rs145497186 in the control and case groups to be GG and AG, respectively. In this study, the minimum allele frequency (MAF) of A was 0.038. The data obtained from the ALFA project showed that the MAF of A in Europeans is 0.0001 and that in Africans is zero. However, in Asians, especially East Asians, the MAF of A is significantly higher, at 0.03. Therefore, rs145497186 associated with A may be a risk factor for LDD in the East Asian population.

According to previous studies, most diseases follow allelic and dominant/recessive model inheritance patterns [43]. The results also showed rs145497186 to be significantly associated with the risk of lumbar disc degeneration in the allelic model, dominant model and heterozygous model (Table 2). In addition, the patients were divided into two groups, VAS < 4 and VAS $\ge$ 4. The results showed that SNP rs145497186, which is related to NDUFV2, was significantly associated with the degree of chronic low back pain, excluding the influence of sex, age, height, weight, BMI, SCOR and DSCR.

Based on LD analysis, the D’ value of rs987850 and rs12965084 was 0.89, and the ${r}^{2}$ value was 0.381. Either a D’ value > 0.8 or a ${r}^{2}$ value > 0.33 indicates that two SNPs are in LD [44]. Therefore, rs987850 and rs12965084 fit well with LD. However, the D’ value of rs145497186 and rs987850 was 0.355 and the ${r}^{2}$ value 0.001. Thus, rs145497186 barely fit with LD, and linkage between them was weak. Overall, the SNP rs145497186 is more likely to act alone.

Using data obtained from NCBI, RegulomeDB, and 3D SNP, the position of rs145497186 is at chr18:9,073,758 (GRCh38.p13), upstream of the NDUFV2 gene; this SNP does not directly affect the function of NDUFV2. The SNP rs145497186 is a noncoding SNP and interacts with NDUFV2 in three-dimensional space through the chromatin loop structure to exert its function. The score for the enhancer was 25.33, suggesting that SNP rs145497186 might increase expression of NDUFV2 by affecting the promoter state, resulting in increased NDUFV2 protein abundance and electron transport chain efficiency. In turn, the generation of free radicals and reactive oxygen species would increase, which might impact the cells of the intervertebral disc and ultimately lead to LDD [45]. In addition, the TFBS score was 100, and the results showed that it is located in a region of 205 transcription factor-binding sites. We speculate that the SNP rs145497186 might affect expression of NDUFV2 via transcription factors. These results all verify that the SNP rs145497186 indirectly, rather than directly, affects the function of NDUFV2.

In summary, we conclude that the SNP rs145497186 related to NDUFV2 could be associated with susceptibility to lumbar disc degeneration and the degree of chronic low back pain in Asian people.

There are still some limitations in this study. For instance, the small sample size is the major limitation of this study, so the discoveries of this study need to be validated on a larger scale in the future study. Although the sample scale is small, the study remains valuable as NDUFV2 rs145497186 has not been studied in LDD and significant conclusion is obtained in this study. In the future large-scale study, we will verify whether this finding can meet the genetic susceptibility of large populations. If the conclusions are consistent, NDUFV2 rs145497186 will be a sensitive SNP site to predict the susceptibility of East Asian populations to LDD. In addition, the specific mechanism by which rs145497186 affects the function of NDUFV2 has not been elucidated. Although a relationship between rs145497186 and LDD has been established, further research will be required to explore the role of the SNP rs145497186 in the pathogenesis of LDD.

## Supplementary Information

**Additional file 1: Table S1.** General data of case and control group.

**Additional file 2: Table S2.** Hardy-Weinberg Equilibrium Test of rs28742109, rs12955018, rs987850, rs8093805, rs12965084 and rs145497186 that related to NDUFV2.

**Additional file 3: Table S3.** Characteristics of Case Group associated with VAS.

## Data Availability

All data generated or analysed during this study were available via contacting the corresponding author.

## Abbreviations

SNPs: Single-nucleotide polymorphisms
LDD: Lumbar disc degeneration
CLBP: Chronic low back pain
NDUFV2: NADH dehydrogenase [ubiquinone] flavoprotein 2
VAS: Visual analogue scale
SCOR: Spinal canal occupation ratio
DSCR: Decrease of spinal canal ratio
BMI: Body mass index
HWE: Hardy–Weinberg equilibrium
LD: Linkage disequilibrium
TFBS: Transcription factor binding sites

## References

[1] Alleva J, Hudgins T, Belous J, Kristin OA (2016). Chronic low back pain. Dis Month.

[2] Kamali A, Ziadlou R, Lang G (2021). Small molecule-based treatment approaches for intervertebral disc degeneration: current options and future directions. Theranostics.

[3] DePalma MJ, Ketchum JM, Saullo T (2011). What is the source of chronic low back pain and does age play a role?. Pain Med.

[4] Brinjikji W, Diehn FE, Jarvik JG (2015). MRI findings of disc degeneration are more prevalent in adults with low back pain than in asymptomatic controls: a systematic review and meta-analysis. AJNR Am J Neuroradiol.

[5] Urano T, Narusawa K, Shiraki M (2011). Single-nucleotide polymorphism in the hyaluronan and proteoglycan link protein 1 (HAPLN1) gene is associated with spinal osteophyte formation and disc degeneration in Japanese women. Eur Spine J.

[6] Risbud MV, Shapiro IM (2014). Role of cytokines in intervertebral disc degeneration: pain and disc content. Nat Rev Rheumatol.

[7] Yang RZ, Xu WN, Zheng HL (2021). Involvement of oxidative stress-induced annulus fibrosus cell and nucleus pulposus cell ferroptosis in intervertebral disc degeneration pathogenesis. J Cell Physiol.

[8] Xie L, Huang W, Fang Z (2019). CircERCC2 ameliorated intervertebral disc degeneration by regulating mitophagy and apoptosis through miR-182–5p/SIRT1 axis. Cell Death Dis.

[9] Kim HJ, Lee HR, Kim H, Do SH (2020). Hypoxia helps maintain nucleus pulposus homeostasis by balancing autophagy and apoptosis. Oxid Med Cell Longev.

[10] Pamplona R, Jové M, Mota-Martorell N, Barja G (2021). Is the NDUFV2 subunit of the hydrophilic complex I domain a key determinant of animal longevity?. Febs J.

[11] Ogura M, Yamaki J, Homma MK, Homma Y (2012). Mitochondrial c-Src regulates cell survival through phosphorylation of respiratory chain components. Biochem J.

[12] Meng Q, Hao Q, Zhao C (2019). The association between collagen gene polymorphisms and intracranial aneurysms: a meta-analysis. Neurosurg Rev.

[13] Aicale R, Tarantino D, Maccauro G, Peretti GM, Maffulli N (2019). Genetics in orthopaedic practice. J Biol Regul Homeost Agents.

[14] Pruna R, Artells R, Lundblad M, Maffulli N (2017). Genetic biomarkers in non-contact muscle injuries in elite soccer players. Knee Surg Sports Traumatol Arthrosc.

[15] Longo UG, Loppini M, Margiotti K (2015). Unravelling the genetic susceptibility to develop ligament and tendon injuries. Curr Stem Cell Res Ther.

[16] Pruna R, Artells R, Ribas J (2013). Single nucleotide polymorphisms associated with non-contact soft tissue injuries in elite professional soccer players: influence on degree of injury and recovery time. BMC Musculoskelet Disord.

[17] Clos E, Pruna R, Lundblad M, Artells R, Maffulli N (2021). ACTN3's R577X single nucleotide polymorphism allele distribution differs significantly in professional football players according to their field position. Med Princ Pract.

[18] Maffulli N, Margiotti K, Longo UG, Loppini M, Fazio VM, Denaro V (2013). The genetics of sports injuries and athletic performance. Muscles Ligaments Tendons J.

[19] Wang Z, Li Y, Wang Y, Wang X, Zhang J, Tian J (2018). Association between GDF5 single nucleotide polymorphism rs143383 and lumbar disc degeneration. Exp Ther Med.

[20] Chen SP, Tsai ST, Jao SW (2006). Single nucleotide polymorphisms of the APC gene and colorectal cancer risk: a case-control study in Taiwan. BMC Cancer.

[21] Luo M, Li JX, Sun XS (2014). The single nucleotide polymorphism rs2208454 confers an increased risk for ischemic stroke: a case-control study. CNS Neurosci Ther.

[22] Vieira LA, Dos Santos AA, Peluso C, Barbosa CP, Bianco B, Rodrigues LMR (2018). Influence of lifestyle characteristics and VDR polymorphisms as risk factors for intervertebral disc degeneration: a case-control study. Eur J Med Res.

[23] Huang LH, Lin PH, Tsai KW (2017). The effects of storage temperature and duration of blood samples on DNA and RNA qualities. PLoS ONE.

[24] Chiarotto A, Maxwell LJ, Ostelo RW, Boers M, Tugwell P, Terwee CB (2019). Measurement properties of visual analogue scale, numeric rating scale, and pain severity subscale of the brief pain inventory in patients with low back pain: a systematic review. J Pain.

[25] Liu H, Hu Y (2010). Hardy-Weinberg equilibrium in genetic epidemiology. Zhong Nan Da Xue Xue Bao Yi Xue Ban.

[26] Gao S, Xun C, Xu T (2021). Associations between vitamin D receptor gene polymorphisms and spinal degenerative disease: evidence from a meta-analysis based on 35 case-control studies. Clin Neurol Neurosurg.

[27] Slatkin M (2008). Linkage disequilibrium–understanding the evolutionary past and mapping the medical future. Nat Rev Genet.

[28] Gerard D (2021). Pairwise linkage disequilibrium estimation for polyploids. Mol Ecol Resour.

[29] Boyle AP, Hong EL, Hariharan M (2012). Annotation of functional variation in personal genomes using RegulomeDB. Genome Res.

[30] Quan C, Ping J, Lu H, Zhou G, Lu Y (2022). 3DSNP 2.0: update and expansion of the noncoding genomic variant annotation database. Nucleic Acids Res.

[31] Lu Y, Quan C, Chen H, Bo X, Zhang C (2017). 3DSNP: a database for linking human noncoding SNPs to their three-dimensional interacting genes. Nucleic Acids Res.

[32] Minelli C, Thompson JR, Abrams KR, Thakkinstian A, Attia J (2008). How should we use information about HWE in the meta-analyses of genetic association studies?. Int J Epidemiol.

[33] Reed MD, Van Nostran W (2014). Assessing pain intensity with the visual analog scale: a plea for uniformity. J Clin Pharmacol.

[34] Yoo SS, Jin C, Jung DK (2015). Putative functional variants of XRCC1 identified by RegulomeDB were not associated with lung cancer risk in a Korean population. Cancer Genet.

[35] Song J, Hao L, Wei W (2020). A SNP in the 3'UTR of the porcine IGF-1 gene interacts with miR-new14 to affect IGF-1 expression, proliferation and apoptosis of PK-15 cells. Domest Anim Endocrinol.

[36] Amini H, Shroff N, Stamova B (2020). Genetic variation contributes to gene expression response in ischemic stroke: an eQTL study. Ann Clin Transl Neurol.

[37] Kishita Y, Shimura M, Kohda M (2021). Genome sequencing and RNA-seq analyses of mitochondrial complex I deficiency revealed Alu insertion-mediated deletion in NDUFV2. Hum Mutat.

[38] Mota-Martorell N, Jove M, Pradas I (2020). Low abundance of NDUFV2 and NDUFS4 subunits of the hydrophilic complex I domain and VDAC1 predicts mammalian longevity. Redox Biol.

[39] Xu C, Li PP, Kennedy JL (2008). Further support for association of the mitochondrial complex I subunit gene NDUFV2 with bipolar disorder. Bipolar Disord.

[40] Nishioka K, Vilariño-Güell C, Cobb SA (2010). Genetic variation of the mitochondrial complex I subunit NDUFV2 and Parkinson's disease. Parkinsonism Relat Disord.

[41] Zhang H, Shao Y, Chen W, Chen X (2021). Identifying mitochondrial-related genes NDUFA10 and NDUFV2 as prognostic markers for prostate cancer through biclustering. Biomed Res Int.

[42] Liu HY, Liao PC, Chuang KT, Kao MC (2011). Mitochondrial targeting of human NADH dehydrogenase (ubiquinone) flavoprotein 2 (NDUFV2) and its association with early-onset hypertrophic cardiomyopathy and encephalopathy. J Biomed Sci.

[43] Zhang H, Chen L, Wang Z (2021). Association of single nucleotide polymorphism rs2228570 with lumbar disc degeneration: a case-control study and meta-analysis. J Pain Res.

[44] Fox EA, Wright AE, Fumagalli M, Vieira FG (2019). ngsLD: evaluating linkage disequilibrium using genotype likelihoods. Bioinformatics.

[45] Bai Z, Liu W, He D (2020). Protective effects of autophagy and NFE2L2 on reactive oxygen species-induced pyroptosis of human nucleus pulposus cells. Aging Albany NY.

