# Supplementary material for: Association between single-nucleotide polymorphism rs145497186 related to NDUFV2 and lumbar disc degeneration: a pilot case–control study
Source: J Orthop Surg Res. 2022 Oct 29;17:473. doi: 10.1186/s13018-022-03368-y (PMC9618206; doi:10.1186/s13018-022-03368-y)
Supplement: Supplementary file 1 — Additional file 1: Table S1. General data of case and control group. [file 13018_2022_3368_MOESM1_ESM.docx]

Table S1. General data of case and control group

| Characteristic | Case group(n=46) | Control group(n=45) | *P* value |
| --- | --- | --- | --- |
| Sex |  |  | 1.00 |
| Male | 22 (47.8%) | 22 (48.9%) |  |
| Female | 24 (52.2%) | 23 (51.1%) |  |
| Age (years) | 50.13$\pm$14.24 | 52.76$\pm$13.73 | 0.37 |
| Height (m) | 1.66$\pm$0.085 | 1.68$\pm$0.095 | 0.36 |
| Weight (Kg) | 70.96$\pm$13.17 | 70.24$\pm$12.49 | 0.79 |
| BMI (Kg/$m^{2}$) | 25.63$\pm$3.77 | 24.88$\pm$3.56 | 0.34 |

*Gender is represented as number and percentage, and the remaining variables are expressed as a mean ± standard deviation; BMI, body mass index.
