# Supplementary material for: Association between single-nucleotide polymorphism rs145497186 related to NDUFV2 and lumbar disc degeneration: a pilot case–control study
Source: J Orthop Surg Res. 2022 Oct 29;17:473. doi: 10.1186/s13018-022-03368-y (PMC9618206; doi:10.1186/s13018-022-03368-y)
Supplement: Supplementary file 2 — Additional file 2: Table S2. Hardy-Weinberg Equilibrium Test of rs28742109, rs12955018, rs987850, rs8093805, rs12965084 and rs145497186 that related to NDUFV2. [file 13018_2022_3368_MOESM2_ESM.docx]

Table S2. Hardy-Weinberg Equilibrium Test of rs28742109, rs12955018, rs987850, rs8093805, rs12965084 and rs145497186 that related to NDUFV2.

| SNP | Control Group (n)^a^ | | | Case Group (n)^a^ | | | P Value |
| --- | --- | --- | --- | --- | --- | --- | --- |
|  | 11 | 12 | 22 | 11 | 12 | 22 |  |
| rs28742109 | 28 | 13 | 4 | 35 | 11 | 0 | 0.692 |
| rs12955018 | 41 | 4 | 0 | 36 | 8 | 2 | 0.238 |
| rs987850 | 26 | 18 | 1 | 36 | 10 | 0 | 0.533 |
| rs8093805 | 18 | 20 | 7 | 31 | 11 | 4 | 0.249 |
| rs12965084 | 18 | 20 | 7 | 29 | 15 | 2 | 0.809 |
| rs145497186 | 45 | 0 | 0 | 39 | 7 | 0 | 0.930 |

*SNP, single nucleotide polymorphism; ^a^11 presents common homozygote, 22 presents rare homozygote, 12 presents heterozygote, 1 presents common allele, 2 presents rare allele.
