# Supplementary material for: Association between single-nucleotide polymorphism rs145497186 related to NDUFV2 and lumbar disc degeneration: a pilot case–control study
Source: J Orthop Surg Res. 2022 Oct 29;17:473. doi: 10.1186/s13018-022-03368-y (PMC9618206; doi:10.1186/s13018-022-03368-y)
Supplement: Supplementary file 3 — Additional file 3: Table S3. Characteristics of Case Group associated with VAS. [file 13018_2022_3368_MOESM3_ESM.docx]

Table S3. Characteristics of Case Group associated with VAS

| Characteristic | VAS<4 (n) | VAS≥4 (n) | *P* value |
| --- | --- | --- | --- |
| Sex (Male/Female) | 8/11 | 14/13 | 0.51 |
| Age (years) | 50.37$\pm$14.56 | 49.96$\pm$14.29 | 0.93 |
| Height (m) | 1.64$\pm0.08$ | 1.67$\pm0.09$ | 0.28 |
| Weight (Kg) | 69.97$\pm$11.55 | 71.65$\pm$14.37 | 0.68 |
| BMI (Kg/$m^{2}$) | 25.90$\pm$4.27 | 25.43$\pm$3.45 | 0.68 |
| SCOR | 0.32$\pm$0.09 | 0.37$\pm$0.13 | 0.20 |
| DSCR | 0.23$\pm$0.08 | 0.22$\pm0.09$ | 0.68 |

*VAS, Visual Analogue Scale; BMI, body mass index; SCOR, spinal canal occupation ratio(SCOR=(b1-b2)/b1×100%, b1 is the anteroposterior diameter of the spinal canal, b2 is the shortest diameter of spinal canal); DSCR, decrease of spinal canal ratio(DSCR=b1/b4-b2/b3, b1 is the anteroposterior diameter of the spinal canal, b2 is the shortest diameter of spinal canal, b3 is the anteroposterior diameter of intervertebral disc, b4 is the anteroposterior diameter of vertebral body).
